# The Role of Gut Microbiome in Prostate Cancer: Current Evidence and Emerging Opportunities

**DOI:** 10.3390/cancers18060998

**Published:** 2026-03-19

**Authors:** Jing Huang, Xin-Hua Zhu, Lloyd C. Trotman, Che-Kai Tsao

**Affiliations:** 1 Tisch Cancer Institute, Icahn School of Medicine at Mount Sinai, New York, NY 10029, USA; 2 Northwell Cancer Institute, Northwell Health, New York, NY 11042, USA; 3 Feinstein Institutes for Medical Research, Northwell Health, New York, NY 11030, USA; 4 Cold Spring Harbor Laboratory, Cold Spring Harbor, NY 11724, USA

**Keywords:** prostate cancer, gut microbiome, dysbiosis, inflammation, androgen deprivation therapy (ADT), short-chain fatty acids (SCFAs)

## Abstract

Prostate cancer (PCa) is one of the most common malignancies in men, and growing evidence implicates the gut microbiome as a significant, modifiable contributor to disease evolution and management. Dysbiosis influences PCa biology through effects on inflammation, immune regulation, metabolism, and hormone signaling. Microbial imbalance can promote systemic inflammation and increase intestinal permeability, activating immune signaling pathways such as NF-κB–IL-6–STAT3. In parallel, microbiome-driven metabolic effects, including IGF-1 signaling and microbial androgen synthesis or recycling, may contribute to resistance to androgen deprivation therapy (ADT). Microbial metabolites, notably short-chain fatty acids (SCFAs) and trimethylamine N-oxide (TMAO), exert context-dependent effects on tumor growth, treatment resistance, and progression. Conversely, beneficial microbes have been associated with improved treatment sensitivity and immune regulation. Together, these insights support the gut microbiome as a potential biomarker and emerging therapeutic target in PCa. Modulation strategies, including diet, probiotics, antibiotics, and fecal microbiota transplantation (FMT), are being explored to improve treatment response and address resistance. As mechanistic evidence continues to grow, ongoing monitoring of the gut microbiome may help inform risk stratification and treatment optimization in prostate cancer.

## Simple Summary

This article explains how the gut microbiome, meaning the bacteria living in our intestines, can affect prostate cancer. When these bacteria are out of balance, they can increase inflammation in the body, weaken the immune system, and produce substances that help cancer grow. Some gut bacteria can even make or recycle male hormones, which can reduce how well common prostate cancer treatments work. On the other hand, healthy gut bacteria may help treatments work better and slow cancer progression. Because gut bacteria are influenced by diet, medications, and lifestyle, changing the microbiome through food choices, supplements, or other therapies may offer new ways to support prostate cancer treatment in the future.

## 1. Introduction

Prostate cancer (PCa) is the second most diagnosed male cancer in the United States. Although most are detected at a localized stage with a favorable prognosis [1,2,3], a subset eventually develops advanced or metastatic disease requiring systemic therapy. Androgen deprivation therapy (ADT) remains a cornerstone of treatment for advanced PCa and typically produces an initial clinical and biochemical response; however, these responses are rarely durable or curative. Most patients ultimately develop castration-resistant prostate cancer (CRPC), a treatment-refractory state associated with limited curative options and responsible for the majority of prostate cancer–related mortality [4,5].

PCa arises through a complex interplay of genetic, hormonal, metabolic, and environmental factors. Among modifiable contributors, the gut microbiome has emerged as an important regulator of systemic metabolism, immune function, and inflammation. Dysbiosis, an imbalance in microbial composition, has been linked to metabolic disorders, chronic inflammation, and multiple cancers [1]. Dysbiosis refers to a pathologic shift in gut microbial composition and function that disrupts host immune and metabolic homeostasis [6]. It often involves reduced diversity, loss of beneficial taxa, and expansion of pro-inflammatory organisms. In prostate cancer, dysbiosis has been associated with reduced microbial richness and alpha diversity, impaired gut barrier integrity, increased systemic translocation of microbial products such as LPS, and activation of inflammatory pathways such as NF-κB IL6 STAT3 [7,8]. In colorectal cancer, for example, dysbiosis promotes carcinogenesis through inflammatory and immune mechanisms [9]. Increasing evidence suggests that similar microbiome-dependent processes may influence PCa risk, progression, and therapeutic response.

**Figure 1:**
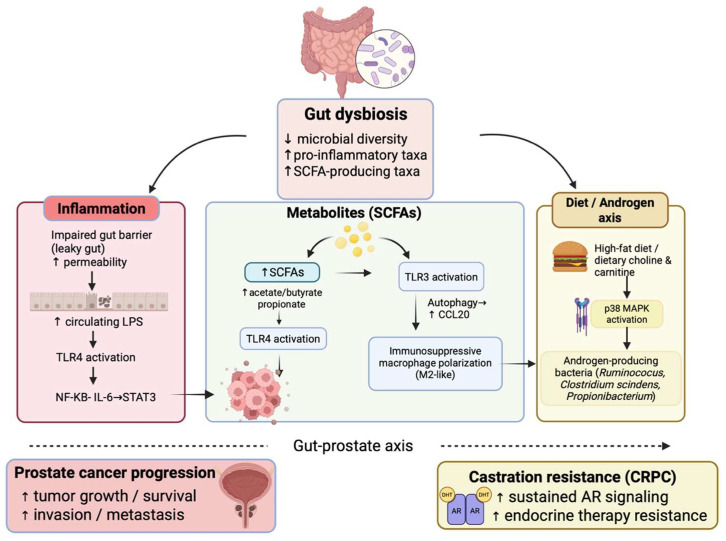
Proposed mechanisms linking gut microbiome dysbiosis to prostate cancer development, progression, and treatment resistance. Created in BioRender. Huang, J. (25 January 2026) https://BioRender.com/kuy61ag (accessed on 31 January 2026). Dysbiosis disrupts gut barrier integrity, enabling systemic translocation of microbial products (e.g., LPS) that activate inflammatory signaling (TLR4–NF-κB–IL-6–STAT3). Microbiome-derived metabolites (SCFAs, TMAO) modulate oncogenic pathways, including IGF-1–PI3K/AKT and p38/HMOX1, while select taxa contribute to androgen biosynthesis and recycling, promoting resistance to androgen deprivation therapy and progression to castration-resistant disease.

Proposed mechanisms include inflammation, hormone metabolism, and microbiome-derived metabolites. TMAO has been associated with increased risk of aggressive prostate cancer and may promote tumor progression through inflammatory and stress-response signaling pathways [10,11,12,13] (Figure 1). Because the microbiome is strongly influenced by diet, medications, lifestyle, and environmental exposures, it represents a modifiable target. Interventions such as dietary modification, probiotics, prebiotics (non-digestible substrates such as dietary fiber that promote beneficial microbial growth), antibiotics, and fecal microbiota transplantation (FMT) are being investigated to reshape the gut–prostate axis and improve PCa outcomes. Therefore, understanding the mechanistic connections between the microbiome and PCa is of great interest as a source of new strategies for prevention, risk assessment, and adjunctive treatment, supporting a more data-driven and individualized approach to PCa management.

### 1.1. The Gut Microbiome: Overview

The gut microbiome comprises bacteria, archaea, fungi, viruses, and protozoa residing in the gastrointestinal tract [11,14]. In healthy adults, dominant bacterial phyla include Firmicutes (e.g., *Faecalibacterium*, *Ruminococcus*, *Clostridium*) and Bacteroidetes (e.g., *Bacteroides*, *Prevotella*), along with contributions from Actinobacteria, Proteobacteria, and Verrucomicrobia [1,5].

These microbes perform essential functions, including fermentation of dietary fibers into short-chain fatty acids, modulation of bile acid composition, and participation in steroid hormone metabolism, including the transformation of androgens and estrogens [15]. Immunologically, the microbiome shapes both local and systemic immunity by influencing T-cell differentiation, balancing pro-inflammatory (Th1, Th17) and anti-inflammatory (Treg) responses, maintaining immune tolerance, and regulating inflammation [6]. It supports epithelial barrier integrity by stimulating mucus production, tight junction protein expression, and antimicrobial peptide release. Regarding microbial diversity, both alpha diversity (within individuals) and beta diversity (between individuals) are key indicators of gut health and resilience [16]. Dysbiosis is characterized by a disruption in diversity or overrepresentation of pro-inflammatory taxa. It has been associated with inflammatory bowel disease, obesity, diabetes, autoimmunity, and cancer [17]. In PCa, distinct microbial compositions and metabolic signatures have been reported, suggesting that the gut microbiome may contribute to oncogenesis and influence treatment responses.

Several recent reviews published in 2024 and 2025 have summarized associations between the gut microbiome and prostate cancer, including microbial composition, metabolic pathways, and immune interactions [18,19,20,21]. This review focuses on emerging mechanistic findings linking microbiome-derived metabolites, inflammatory signaling pathways, and microbial androgen metabolism to prostate cancer tumor progression and endocrine therapy resistance, while highlighting ongoing clinical trials.

### 1.2. Intraprostatic Microbiome and Prostate Cancer

Although this review focuses primarily on the gut microbiome, emerging evidence indicates that the prostate is not sterile and may harbor a distinct intraprostatic microbial community. Sequencing-based studies have identified bacterial taxa within prostate tissue, urine, and prostatic secretions, with potential links to inflammation and carcinogenesis [18,19]. These findings suggest that local microbial communities may contribute to prostate cancer biology and interact with systemic microbiota within a broader gut–prostate axis. However, a comprehensive discussion of the intraprostatic microbiome is beyond the primary scope of this review, and the following sections therefore emphasize gut microbiome-mediated mechanisms.

### 1.3. Microbiome Composition of PCa Patients

Controlled animal models support the concept that androgen deprivation alters feeding behavior and systemic metabolism. In orchidectomized (ORX) and androgen receptor knockout (ARKO) mice, androgen deficiency reduces food intake yet paradoxically increases adiposity and worsens glucose homeostasis, particularly under high-fat diet conditions. These findings suggest that ADT may indirectly reshape the gut environment through changes in diet patterns, energy balance, and metabolic signaling, providing a rationale for investigating microbiome shifts in prostate cancer [22,23]. Consistent with this host metabolic shift, the gut microbiome of men with PCa differs significantly from that of healthy individuals and benign prostatic hyperplasia (BPH) controls with respect to microbial composition and overall diversity. Several studies report alterations in both bacterial composition and microbial metabolic capacity. Golombos et al. (2018) identified higher levels of *Bacteroides massiliensis* in PCa patients, whereas BPH controls demonstrated enrichment of beneficial metabolite-producing genera such as *Faecalibacterium* and *Eubacterium* [24]. Liss et al. (2018) further showed that PCa patients exhibit reduced folate biosynthetic capacity, suggesting alterations in essential microbial metabolic pathways [25]. Distinct patterns also appear to correlate with disease stage and aggressiveness. The genus *Ruminococcus*, which is involved in SCFA production and androgen metabolism, is consistently enriched in advanced disease, including castration-resistant prostate cancer (CRPC) [26,27]. Additional taxa associated with high-risk or high-Gleason grade PCa include *Alistipes*, Rikenellaceae, and *Lachnospira* [28]. Increased abundance of Proteobacteria, a phylum often considered a marker of inflammation and dysbiosis, has been strongly linked to metastatic disease and correlates with nodal and distant spread [1,8], suggesting its potential utility as a biomarker of disease progression. Similarly, some studies have identified a higher abundance of Firmicutes (Bacillota), particularly classes such as Bacilli and Erysipelotrichia, with corresponding shifts in the Firmicutes-to-Bacteroidetes ratio in PCa patients [3,5,29]. Therapeutic interventions also shape microbiome composition. Pernigoni et al. (2021, 2023) demonstrated that ADT promotes the expansion of *Ruminococcus* species capable of androgen biosynthesis, thereby contributing to resistance and progression to CRPC [30,31].

Preclinical animal models provide strong evidence that specific gut microbiota taxa can actively drive PCa progression across distinct disease stages, with causality supported by microbial transplantation, supplementation, and depletion studies [5,6,29]. In the transition from hormone-sensitive prostate cancer (HSPC) to castration-resistant prostate cancer (CRPC), androgen-producing taxa appear to play a central role, particularly *Ruminococcus* species [32]. In mouse models, fecal microbiota transplantation (FMT) from CRPC patients enriches taxa including *Ruminococcus* and accelerates tumor progression, while depletion of these organisms with antibiotics delays castration resistance. Oral supplementation with *Ruminococcus gnavus* promotes CRPC progression, and these effects are transmissible via FMT, supporting a causal role for specific microbes in androgen-refractory disease [6,27,33]. Mechanistically, *R. gnavus* expresses CYP17A1-like enzymes capable of converting pregnenolone into dehydroepiandrosterone (DHEA) and testosterone, providing an alternative androgen source that may fuel CRPC development [6].

Beyond androgen metabolism, pro-inflammatory and genotoxic taxa may contribute to early carcinogenesis. Infection with *Helicobacter hepaticus* in genetically susceptible ApcMin/+ mice enhances prostatic intraepithelial neoplasia (PIN) and microinvasive carcinoma in an inflammation-dependent manner, and neutralization of TNF-α prevents cancer transmission, demonstrating that systemic inflammation induced by specific gut bacteria can promote neoplastic progression [34]. Diet–microbiome interactions also influence disease behavior. In PTEN-null and TRAMP models, high-fat diet–associated enrichment of short-chain fatty acid-producing taxa has been linked to increased IGF-1 signaling through MAPK and PI3K pathways and accelerated tumor growth, while antibiotic treatment can attenuate these effects [35,36].

Clinical studies are generally consistent with the presence of microbiome differences in PCa and during treatment, although results vary across cohorts and are subject to confounding from diet, medications, comorbidities, and disease stage. For example, Kure et al. (2023) reported that ADT was associated with reduced microbial diversity and shifts in taxa, including enrichment of *Ruminococcus* and Bacteroides [37]. While these findings are hypothesis-generating, additional longitudinal studies and mechanistic validation are needed to determine whether specific taxa directly modulate therapeutic response or primarily reflect underlying host metabolic status. Conversely, some taxa may exert protective effects in preclinical settings. *A. muciniphila* has been associated with improved treatment sensitivity and delayed progression in mouse models, where oral administration enhanced ADT efficacy and increased tumor-infiltrating T cells. Its metabolite inosine reduced intestinal barrier damage and lipopolysaccharide translocation, limiting downstream inflammatory signaling implicated in disease progression [38]. Together, these preclinical data support a functional gut–prostate axis and provide a mechanistic framework for interpreting human microbiome associations.

Beyond treatment effects, multiple studies affirm that butyrate-producing genera like *F. prausnitzii* and *Eubacterium rectale*, known for their anti-inflammatory and epithelial health-promoting effects, are significantly depleted in PCa patients compared to both healthy and BPH controls. This consistent finding across various cohorts suggests a loss of beneficial SCFA-mediated metabolic functions in PCa patients [15,39,40]. Nevertheless, the degree of depletion varies across cohorts, raising the possibility of geographic, dietary, and methodological influences [32,39].

Microbial diversity represents an important indicator of gut ecosystem health. Alpha diversity refers to the richness and evenness of microbial taxa within an individual sample, whereas beta diversity describes differences in microbial community composition between individuals or groups [6]. While several studies report significant alpha diversity reductions in patients with greater tumor burden or more aggressive disease, others observe no differences, suggesting that additional host or environmental factors may confound these findings. In contrast, beta diversity consistently distinguishes PCa from non-cancer cohorts across studies, reinforcing the presence of disease-specific microbial signatures despite variability in individual taxa [33]. Taken together, these clinical observations indicate that PCa is associated with measurable microbiome differences. However, the extent to which diversity changes directly influence disease progression or treatment response remains uncertain and requires mechanistic validation.

Preclinical transplantation and supplementation studies support a functional role for the gut microbiome in prostate cancer progression and treatment response. FMT experiments demonstrate that microbiota derived from aggressive or castration-resistant disease can accelerate tumor growth and resistance phenotypes in recipient models, whereas microbiota from healthy or treatment-responsive donors may enhance tumor control and androgen deprivation therapy sensitivity [4,27,29]. Mechanistically, these effects may involve microbiome-derived metabolites, inflammatory signaling pathways, and microbial androgen metabolism, which are discussed in greater detail in Section 2.

## 2. Evidence Linking the Gut Microbiome to Prostate Cancer

### 2.1. Microbial Metabolites

Growing evidence suggests that microbiome-derived metabolites, particularly short-chain fatty acids (SCFAs), may influence prostate cancer biology through metabolic and immune mechanisms [1,2,3]. Experimental models indicate that alterations in SCFA-producing microbial communities can modulate systemic and intraprostatic signaling pathways, including IGF-1–associated PI3K and MAPK activation, with downstream effects on tumor proliferation and therapeutic response [1,4,41]. Nevertheless, these findings are largely derived from preclinical studies, and the clinical relevance of SCFA-mediated signaling in prostate cancer remains incompletely defined [2,5].

By contrast, clinical efforts to inhibit IGF-1 signaling directly have produced limited benefit. Cixutumumab (IMC-A12), a recombinant fully human IgG1 anti-IGF-1R monoclonal antibody, modestly increased rates of undetectable PSA (≤0.2 ng/mL) when added to androgen deprivation therapy in newly metastatic hormone-sensitive disease but did not improve overall survival or progression outcomes in SWOG S0925 [42]. Likewise, figitumumab (CP-751,871), a human IgG2 anti-IGF-1R antibody, failed to improve outcomes in metastatic castration resistant prostate cancer and was associated with shorter progression-free survival and greater serious toxicity when combined with docetaxel versus docetaxel alone [43]. Together, these results suggest that direct IGF-1R blockade may be insufficient, and that upstream modulation of the gut microbiome and its metabolite-driven effects on IGF-1 signaling warrants further mechanistic and biomarker-guided evaluation.

Beyond IGF-1–associated signaling, SCFAs may influence prostate cancer through immune modulation, autophagy-related pathways, and tumor microenvironment remodeling, with preclinical studies linking these mechanisms to macrophage polarization and disease aggressiveness; however, these observations remain largely model-derived and require clinical validation [7,15,27]. In addition to SCFAs, trimethylamine N-oxide (TMAO), a microbiota-derived metabolite generated from dietary choline, L-carnitine, and betaine, has been implicated in PCa progression [7,12]. Elevated TMAO promotes tumor growth and metastasis through p38 MAPK activation and HMOX1 upregulation, and HMOX1 inhibition reverses these effects [7]. Epidemiologic associations remain mixed, with a metabolomic analysis of the ATBC study [44,45] linking higher TMAO to aggressive PCa and PLCO showing no association with lethal disease [24,34].

Broad-spectrum antibiotics further reshape tumor biology by inducing dysbiosis. Across multiple malignancies, antibiotic exposure near immune checkpoint inhibitor initiation is associated with reduced response rates and shorter progression-free and overall survival, consistent with impaired microbiome-supported anti-tumor immunity [32,35,36,38,46]. Mechanistically, reduced MAdCAM-1 can promote trafficking of Tregs from the gut to peripheral tumors, reinforcing an immunosuppressive microenvironment and limiting checkpoint inhibitor activity [35,47,48,49]. Of interest, a recent study suggests that recovery times for regaining microbial diversity after antibiotics are significantly lengthened in animal models consuming a western high-fat diet [50]. In addition to this qualitative change, a microbial monopolization was also observed, and intriguingly, this dysbiosis was most effectively counteracted by dietary intervention.

Notably, antibiotics can have context-dependent effects in PCa models. In high-fat diet Pten-knockout mice, antibiotics reduce SCFA-producing taxa and suppress systemic and intraprostatic IGF-1, attenuating PI3K and MAPK signaling and slowing tumor growth, while SCFA reconstitution restores the tumor-promoting phenotype [51]. However, because broad depletion can also impair immune competence, selective microbiome modulation and quantitative monitoring will likely be necessary to translate these findings into safe therapeutic strategies [52].

### 2.2. Inflammation

Chronic inflammation is a well-established driver of prostate cancer initiation and progression, and the gut microbiome contributes to this inflammatory milieu through dysbiosis. Disruption of the microbial ecosystem often results in overgrowth of gram-negative bacteria such as Proteobacteria, whose outer membranes contain LPS. This imbalance compromises intestinal barrier integrity by reducing tight junction proteins, including zona occludens-1, leading to increased intestinal permeability or “leaky gut” [5,7,8]. As permeability rises, LPS enters the circulation in a process known as metabolic endotoxemia and can accumulate in distant tissues, including the prostate tumor microenvironment (TME) [1,7,8].

Within the TME, LPS promotes inflammation primarily through activation of the NFκB–IL6–STAT3 pathway [8,33]. LPS binds to Toll-like Receptor 4 (TLR4) on prostate cancer and immune cells, triggering NFκB activation and secretion of pro-inflammatory cytokines such as IL-6, TNF-α, and TGF-β [8,13]. These cytokines subsequently activate STAT3, which upregulates genes that drive cellular proliferation, enhance metastatic potential, and facilitate malignant transformation. Activation of this axis also contributes to therapeutic resistance, including reduced sensitivity to docetaxel and the promotion of castration resistance through ligand-independent androgen receptor signaling [51].

### 2.3. Antibiotic Use, Genotoxicity, and Epigenetics

Antibiotic-induced dysbiosis can promote immune evasion and inflammation by depleting commensal bacteria, reducing microbial diversity, impairing antigen presentation, and weakening effector T-cell function. This is particularly relevant in prostate cancer, which is historically resistant to immunotherapy. In Pten-deficient mouse models, broad-spectrum antibiotics enrich inflammatory Proteobacteria and impair gut barrier function, increasing systemic LPS translocation. Elevated LPS levels activate the NFκB–IL6–STAT3 axis in the TME, enhancing tumor proliferation and conferring docetaxel resistance [6,31,32]. Dysbiosis also diminishes androgen deprivation therapy efficacy, augments androgen receptor signaling, and reduces neutrophil and T-cell–mediated tumor surveillance [23,46]. Although some studies show delayed castration resistance due to depletion of androgen-synthesizing bacteria, these benefits may be offset by reduced microbial diversity and immune competence, supporting the need for patient-specific microbiome profiling [35].

The microbiome also contributes to prostate carcinogenesis through genotoxic mechanisms. Certain Escherichia coli strains produce colibactin, a genotoxin that induces DNA double-strand breaks and chromosomal rearrangements in prostate epithelial cells [2,13]. Microbial-induced genomic damage has been linked to key prostate cancer–associated alterations, including the TMPRSS2–ERG fusion, underscoring the mutagenic potential of specific gut microbes [13]. Beyond inflammation and genotoxicity, the gut microbiome influences epigenetic regulation and metabolic signaling. Obesity-associated dysbiosis has been tied to elevated IGF-1 levels, increased immune cell infiltration, and heightened cytokine production in the prostate, collectively promoting tumor growth and therapeutic resistance [7]. Together, these findings highlight the multifactorial role of the gut microbiome in shaping inflammatory responses, genomic instability, and treatment resistance in prostate cancer.

### 2.4. Hormone Metabolism and the Androgen Signaling Axis

The gut microbiome exerts a significant influence on androgen regulation and prostate cancer progression, particularly in the context of ADT. Certain commensal bacteria, including *Ruminococcus* species, *Clostridium scindens,* and *Propionibacterium* express enzymes capable of performing steroidogenesis analogous to human CYP17A1. These microbes can convert precursors such as pregnenolone into testosterone and dihydrotestosterone (DHT), providing an alternative source of androgens to sustain tumor growth during ADT [4,30]. This localized androgen production undermines the therapeutic intent of ADT, which suppresses androgen synthesis in the testes and adrenal glands, by providing prostate cancer cells with an alternative supply of ligands for the androgen receptor (AR). The microbiota also participates in the deglucuronidation of conjugated steroid hormones, allowing reactivation and reabsorption of testosterone and DHT that were otherwise destined for excretion. Studies have shown correlations between microbial composition and serum testosterone levels, further supporting a role for the gut microbiome in systemic androgen regulation, such as an association between increased Firmicutes abundance and higher serum testosterone in men [9,53,54,55].

ADT reshapes the gut microbiome in ways that establish a self-reinforcing cycle of therapeutic resistance. Evidence from both murine and human studies shows that androgen deprivation therapy enriches androgen-producing bacteria, particularly *Ruminococcus* and related Ruminococcaceae, thereby expanding microbial populations capable of sustaining tumor growth under androgen-limited conditions. This feedback loop accelerates the progression from HSPC to CRPC, a more aggressive and treatment-refractory state [6,31].

Preclinical data support these mechanisms. In mouse models, broad-spectrum antibiotics that depleted the gut microbiota delayed CRPC onset and enhanced the efficacy of ADT, indicating that microbial activity is required for resistance to develop. However, as discussed previously, these effects likely reflect non-specific depletion of many microbial taxa. Fecal microbiota transplantation studies provide more targeted evidence: microbiota from CRPC patients accelerated tumor growth and induced castration resistance in mice, whereas transplants from healthy donors or HSPC patients restored therapeutic sensitivity and slowed tumor progression [5]. Human studies likewise show consistent compositional differences between HSPC and CRPC patients, with the CRPC microbiome enriched for androgen-producing and SCFA-producing taxa such as Ruminococcus, Phascolarctobacterium, and Alistipes [26].

In addition to direct microbial androgen biosynthesis, several indirect mechanisms reinforce endocrine resistance. These include activation of pro-inflammatory pathways such as NF-κB via LPS, modulation of the tumor microenvironment by SCFAs, and interactions between the gut microbiome and prostate cancer therapies [8,56]. For example, treatment with abiraterone acetate selectively enriches *A. muciniphila* [4]. Although the implications remain under investigation, some studies suggest that this organism may exert context-dependent, potentially beneficial effects, underscoring that microbiome drug interactions are complex and therapy-specific [51].

## 3. Therapeutic Opportunities: Targeting the Gut Microbiome

**Figure 2:**
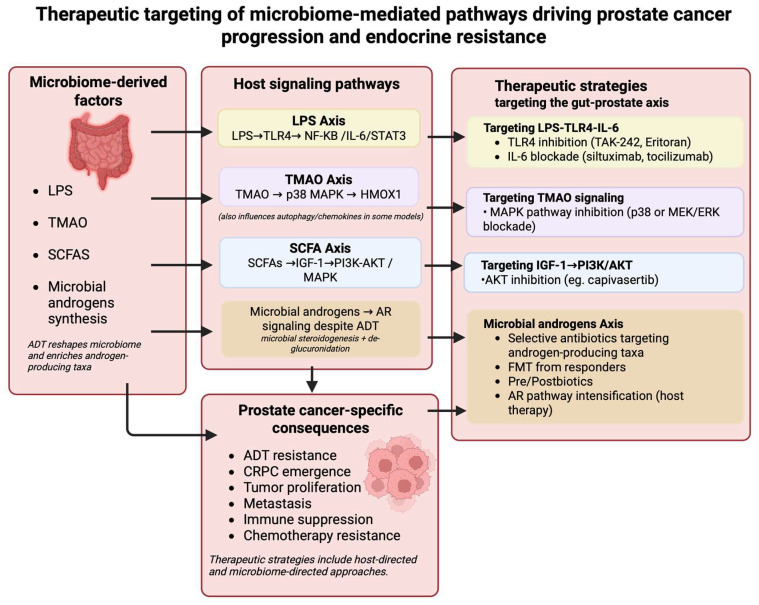
Microbiome-directed therapeutic strategies targeting the gut–prostate axis in prostate cancer. Created in BioRender. Huang, J. (26 February 2026) https://BioRender.com/ej7lufq (accessed on 26 February 2026). Dietary interventions, probiotics/synbiotics, antibiotics, postbiotics, and fecal microbiota transplantation aim to reshape microbial composition and function to reduce inflammation, modulate metabolite signaling, limit androgen-producing taxa, and enhance anti-tumor immunity. These approaches are being evaluated as adjunctive strategies to improve treatment sensitivity and delay progression in prostate cancer. Different colors are used to help distinguish microbiome-derived factors, host signaling pathways, and therapeutic strategies.

There is increasing interest in strategies that modify the gut microbiome to influence prostate cancer development, progression, and treatment responsiveness. These approaches include dietary interventions, probiotics, prebiotics, antibiotics, and fecal microbiota transplantation. The rationale for these therapies is supported by both mechanistic insights and early clinical studies. While it is known that a Western high-fat diet favors microbial monopolies and dysbiosis, a recent preclinical study suggests that dietary interventions are superior to FMT for restoring microbial diversity after antibiotic depletion [50]. Ongoing and planned clinical trials are evaluating how these interventions alter microbial composition, metabolite profiles, inflammation, androgen signaling, and treatment outcomes in prostate cancer. The overall goal is to determine whether the microbiome can be shaped to reduce tumor growth, overcome resistance, and improve long-term disease control. Figure 2 summarizes the major therapeutic strategies currently under investigation, and Table 1 outlines active clinical trials in this area.

### 3.1. Dietary Interventions

Diet is a key modulator of the gut microbiome and has significant implications for prostate cancer biology. Dietary patterns influence microbial composition, intestinal barrier function, and production of metabolites such as SCFAs and TMAO. These changes affect inflammation, androgen signaling, and oncogenic pathways in the prostate [1].

High-fat diets (HFD) and saturated fats are among the most consistent dietary risk factors. A Western-style diet rich in animal-derived saturated fats induces gut dysbiosis and compromises intestinal barrier integrity, allowing bacterial products such as lipopolysaccharide (LPS) to enter circulation and trigger systemic inflammation that promotes tumor growth [5,8,29]. Furthermore, several preclinical studies reveal the importance of prebiotics in the form of fiber/complex carbohydrates for regenerating the microbial community after antibiotics. This has been shown in both high and low-fat diet contexts [50,57,58,59].

Mouse studies show that lard-based HFDs enrich Clostridiales while reducing beneficial Lactobacillales, increasing circulating IGF-1, and activating proliferative PI3K and MAPK pathways [6,60]. Matsushita et al. (2021) confirmed that HFD accelerated tumor growth in Pten-knockout mice, an effect reversed by antibiotics that reduced Rikenellaceae and Clostridiales abundance, lowered SCFA and IGF-1 levels, and suppressed tumor proliferation [28]. Similarly, Zhong et al. (2022) showed that antibiotic-induced dysbiosis enriched Proteobacteria, increased gut permeability, and elevated intratumoral LPS, activating the NF-κB–IL6–STAT3 axis to drive PCa progression and chemotherapy resistance [8].

In contrast, omega-3 polyunsaturated fatty acids exert protective effects. Long-chain omega-3s modulate microbial composition and reduce metabolites linked to PCa aggressiveness [6]. Lachance et al. 2024 demonstrated that dietary supplementation with the omega-3 derivative MAG-EPA reduced the abundance of Ruminococcaceae and fecal butyrate levels in both mice and men with PCa [29]. Elevated butyrate was otherwise correlated with tumor aggressiveness and burden, highlighting its dual context-dependent role [35]. Importantly, omega-3 supplementation reduced tumor growth in mouse models and, in a human pre-prostatectomy trial, was associated with reduced rates of cancer upgrading at surgery [29,61,62]. Current clinical trials are investigating this potential. For example, Mediterranean diet trials (Table 1, NCT05590624 and NCT04985565) are exploring whether a high-fiber Mediterranean diet can alter gut microbiota and systemic biomarkers in men with localized PCa, with the aim of reducing tumor aggressiveness before surgery. Another study (NCT03087903) is evaluating grape seed extract as a prebiotic to beneficially alter the gut microbiome and slow PSA rise in men with asymptomatic, non-metastatic PCa. Personalized lifestyle interventions combining diet and physical activity are being studied in metastatic patients undergoing androgen deprivation therapy (NCT05850182), while early-phase work is assessing acetate supplementation via apple cider vinegar (NCT05802121) to explore SCFAs as modulators of immune function and anti-tumor responses [6].

By contrast, high-choline diets, common in red meat and eggs, are linked to increased risk of lethal PCa through gut microbe-dependent pathways. Specific bacteria metabolize choline into trimethylamine (TMA), which is converted in the liver to trimethylamine N-oxide (TMAO), a metabolite implicated in cardiovascular and cancer risk [13]. Prospective analyses from the PLCO Cancer Screening Trial revealed that elevated plasma choline, betaine, and phenylacetylglutamine were associated with a two- to three-fold increase in lethal PCa [34]. Mechanistic studies show that TMAO directly enhances proliferation and migration of PCa cells via the p38/HMOX1 pathway, providing a molecular link between diet, microbiota activity, and tumor progression [12,13].

Finally, dietary fiber and plant-based diets, particularly the Mediterranean diet, support a healthier gut ecosystem and may offer anti-cancer benefits. Fermentable fibers promote beneficial taxa such as Bifidobacterium and increase SCFA production, creating an anti-inflammatory gut environment and bolstering immune function [63,64]. Preclinical studies demonstrate that high-fiber diets enhance anti-tumor immunity, though human data remain mixed. One intervention study in overweight and obese PCa patients found that dietary modification altered gut microbiota composition, with changes in fat and fiber intake correlating with shifts in Blautia [1,24]. However, larger clinical studies of the Mediterranean diet have not consistently demonstrated protective effects, reflecting the complexity of translating preclinical benefits to patient populations.

### 3.2. Probiotics, Prebiotics and Postbiotics

Probiotics and prebiotics are emerging as promising strategies to modulate the gut microbiome in ways that support prostate health and improve cancer outcomes. By influencing the gut–prostate axis, these interventions can alter inflammation, oxidative stress, and immune surveillance, thereby shaping prostate cancer progression.

Probiotics, defined as live microorganisms that confer health benefits, help restore microbial balance, reduce inflammation, and enhance anti-tumor immunity [17]. They compete with pathogenic species, strengthen intestinal barrier function, and limit systemic exposure to inflammatory molecules such as LPS. Probiotics also modulate host immunity by stimulating mucosal IgA production, regulating T-cell activity, and promoting cytokines with anti-cancer effects. Specific strains show notable immunomodulatory properties: *Lactobacillus* enhances natural killer cell function, while *Bifidobacterium* improves dendritic cell activity and promotes CD8+ T-cell recruitment, augmenting response to immune checkpoint inhibitors [17]. *A. muciniphila* has been associated with stronger anti-tumor immune responses and improved sensitivity to ADT, and *Clostridium* butyricum is currently being evaluated in immunotherapy trials [17,24].

Live biotherapeutics extend this concept through the administration of defined bacterial strains with known immunomodulatory effects. In preclinical models, oral supplementation with *A. muciniphila* improves ADT and PD-1 or PD-L1 inhibitor responses by inducing a Th1 immune profile [65], *Bifidobacterium* enhances anti-PD-L1 therapy [66], and Bacteroides fragilis has been shown to augment anti-CTLA-4 activity [67,68]. MRx0518 (NCT03934827), a live biotherapeutic product, is currently under clinical investigation in patients with solid tumors, including prostate cancer, to evaluate safety and its ability to beneficially modulate the tumor immune microenvironment prior to surgery.

Prebiotics, which are non-digestible dietary substrates that selectively stimulate beneficial microbes, also play an important role in shaping the gut–prostate axis [6]. Dietary fiber supports beneficial taxa such as *Lactobacillus* and *Bifidobacterium* and promotes SCFA production, which maintains gut barrier integrity and regulates immune function. The role of SCFAs is context dependent, as butyrate has been associated with advanced prostate cancer in certain settings. Isoflavones from soy products have shown anti-tumor effects in preclinical studies, driven largely by microbial conversion to equol [13,24].

A recent study by Thomas et al. (2025) compared a phytochemical-rich supplement (PRS) with a combined synbiotic regimen (PRS plus pre/probiotic blend (PB)) in men with rising PSA [69]. PRS alone significantly slowed PSA progression, reducing the average increase from 19.6 percent to 6.2 percent. The combined PRS plus PB therapy produced substantially greater benefit, shifting PSA trajectories from a 21.7 percent increase to a 20 percent decrease, a net improvement of 41.7 percent and a 28.3 percent greater effect compared with PRS alone. Mechanistic analyses suggest that PRS provides antioxidant and anti-inflammatory effects and supports beneficial microbial growth, whereas the probiotic and prebiotic blend enhances microbial balance, gut barrier function, systemic inflammation, and immune surveillance. Together, these synergistic actions strengthen gut microbiome resilience and reduce tumor-promoting processes [69].

Postbiotics are defined as metabolites of probiotic bacterial microorganisms or nonliving bacterial products and/or cells. The potential role of postbiotics in PCa is an emerging area of preclinical research, with studies largely focusing on cytotoxic and immunomodulatory effects [70]. In vitro, postbiotics derived from heat-killed *Lactobacillus acidophilus* LA-5 have demonstrated dose-dependent cytotoxicity in PCa cell lines, including LNCaP and PC3 [71]. These effects appear to involve activation of the intrinsic mitochondrial apoptotic pathway, with increased expression of proapoptotic markers such as Bax, cytochrome c, caspase-9, and caspase-3, alongside suppression of the antiapoptotic protein Bcl-2 [71]. At higher concentrations, these postbiotics also increase oxidative stress markers, including reactive oxygen species (ROS) and malondialdehyde (MDA), while reducing endogenous antioxidant defenses, suggesting a mechanism that may promote apoptosis in malignant cells [71].

In contrast, the most extensively studied postbiotics, short-chain fatty acids (SCFAs), may exert context-dependent effects in PCa. While SCFAs have been widely described as anti-inflammatory and anti-proliferative, more recent evidence has linked elevated SCFA levels with high-grade and castration-resistant prostate cancer (CRPC) [72]. Proposed mechanisms include SCFA-associated increases in IGF-1 signaling through MAPK and PI3K pathways, induction of cancer cell autophagy, and polarization of macrophages toward an anti-inflammatory phenotype within the tumor microenvironment [48,73,74]. As adjunctive therapeutic agents, postbiotics offer practical advantages over live probiotics, including greater stability and improved safety profiles, which may be particularly relevant for immunocompromised patients [75,76]. Their reported biological effects include strengthening intestinal barrier integrity, inhibiting NF-κB signaling, and supporting immune homeostasis [32,70]. Although direct clinical evidence supporting postbiotic use in PCa remains limited, this strategy aims to leverage microbial-derived compounds to modulate inflammation and immune responses and potentially enhance the effectiveness of conventional therapies. Importantly, postbiotic approaches should be selective, prioritizing metabolites that support barrier function and immune regulation while avoiding or limiting metabolites that may promote tumor growth or therapeutic resistance in specific contexts.

An intriguing example of an anti-cancer postbiotic small molecule is the case of menadione, because it happens to be a cell essential for both human and bacterial cells. Our preclinical trials showed that as an oral supplement, menadione (aka vitamin K3) is able to suppress prostate cancer through an oxidative cell death, which we termed triaptosis [77,78]. In mammals, menadione is the precursor of the essential micronutrient vitamin K2. But at the same time, a large fraction of our microbiome depends on vitamin K2 as an electron carrier in the respiratory chain. It remains to be tested if a symbiotic relationship around vitamin K2 metabolism could supply the body’s circulation with the anti-cancer pro-oxidant menadione (vitamin K3).

Despite these encouraging findings, challenges remain. Probiotic effects are highly strained and host-specific, and much of the strongest evidence for microbiome-mediated immunomodulation comes from melanoma, colorectal cancer, or broader immunotherapy studies rather than from prostate cancer research [29]. Well-designed prostate cancer–focused clinical trials are needed to establish efficacy, refine symbiotic formulations, and support translation into clinical practice.

### 3.3. Antibiotics as Microbiome Modulators

Although antibiotics consistently induce gut dysbiosis, their effects on prostate cancer range from harmful to, in specific contexts, potentially beneficial [29,35]. Most evidence highlights the detrimental effects of broad-spectrum antibiotics in prostate cancer, as they can disrupt the gut microbiome, increase inflammation, and reduce the efficacy of treatments such as androgen deprivation therapy (ADT), docetaxel, and immune checkpoint inhibitors, as discussed earlier. These harmful effects are largely driven by antibiotic-induced dysbiosis, enrichment of pro-inflammatory bacteria, and activation of LPS-mediated signaling pathways [4,35]. However, context-dependent benefits have also been reported. Pernigoni et al. (2021) demonstrated that certain gut bacteria, particularly Ruminococcus, can synthesize androgens from precursors and promote resistance to ADT, with antibiotic-mediated depletion delaying castration resistance in mice [30]. In high-fat diet models, antibiotics inhibited tumor proliferation by reducing short-chain fatty acid production, which otherwise promoted cancer growth via IGF-1 signaling [4,35]. Clinically, one study in chemotherapy-naïve metastatic castration-resistant prostate cancer patients found that prolonged antibiotic use during oral systemic therapy correlated with longer overall and progression-free survival, although the underlying mechanisms remain unclear [79]. Together, these findings suggest that while broad-spectrum antibiotics are a blunt tool that often worsens prostate cancer outcomes, targeted elimination of specific androgen-producing taxa may provide therapeutic benefit, a concept currently being explored in ongoing clinical trials combining antibiotics with androgen-axis therapies [6].

### 3.4. Fecal Microbiota Transplantation (FMT)

Fecal microbiota transplantation (FMT) is a promising but still experimental strategy for modifying the gut microbiome in prostate cancer. FMT from patients with prostate cancer or castration-resistant prostate cancer accelerates tumor growth in mice and promotes castration resistance. In contrast, FMT from healthy donors or from patients with hormone-sensitive disease slows tumor progression and enhances the efficacy of androgen deprivation therapy [11,29].

FMT has also been shown to reverse tumor-promoting effects induced by antibiotics and to reduce the abundance of SCFA-producing taxa such as *Ruminococcus* and Alistipes [27,29]. These findings support the concept that a healthy microbiome can restrain tumor growth by reducing tumor-supportive metabolites and inflammatory mediators. Clinical investigation of FMT in PCa is at an early stage. A phase II trial (NCT04116775) is testing FMT in combination with pembrolizumab and enzalutamide in men with metastatic CRPC to evaluate whether treatment resistance can be reversed and PSA decline achieved [6]. While results are pending, other cancers provide encouraging proof-of-concept. In melanoma, FMT from anti–PD-1 responders have overcome immunotherapy resistance, yielding partial and complete tumor responses [5,24,80]. In gastroesophageal cancer, allogenic FMT improved disease control and overall survival compared with autologous FMT [81,82]. Preclinical models in pancreatic cancer also suggest FMT can enhance anti-tumor immunity [17]. Moreover, FMT has been shown to lower microbiome-derived PCa risk scores in high-risk individuals, hinting at possible preventive applications [83]. The rationale is that transferring gut microbiota from a healthy donor or therapy responder could restore a beneficial microbial environment, re-sensitizing the tumor to treatment.

Clinical translation faces important challenges. FMT is generally well tolerated, with most adverse events being mild gastrointestinal symptoms, but rare serious complications such as bacteremia and sepsis underscore the need for strict donor screening [84,85]. Options such as colonoscopy, enema, oral capsules, and nasoduodenal tubes each carry trade-offs in efficacy, invasiveness, and patient acceptability [5].

## 4. Current Challenges and Future Directions

The future of gut microbiome–mediated interventions in prostate cancer will depend on shifting from generalized approaches to personalized strategies informed by robust baseline characterization. Comprehensive microbial and metabolomic profiling can stratify patients, predict treatment responses, and guide targeted interventions. Specific taxa show strong predictive value: *A. muciniphila* correlates with improved responses to immunotherapy and androgen deprivation therapy, whereas enrichment of Proteobacteria and reduced microbial diversity are associated with aggressive disease [6]. Metabolites provide additional resolution, with choline derivatives and phenylacetylglutamine linked to higher risk, and inosine, a metabolite produced by *A. muciniphila*, associated with durable treatment benefit [3,51]. These observations highlight the potential of microbiome-derived biomarkers to complement traditional indicators such as PSA.

Baseline testing strategies continue to expand. Reduced alpha diversity is associated with poor outcomes across multiple malignancies, including melanoma, lung, prostate, and pancreatic cancers, while beta diversity distinctions help differentiate healthy, at-risk, and diseased populations. Screening for favorable taxa such as *F.prausnitzii* or *Bifidobacterium* and unfavorable markers such as *Ruminococcus* or Proteobacteria can support risk stratification. Key microbial metabolites, including SCFAs, TMAO, and inosine, add prognostic value, and profiling microbial capacity for androgen synthesis may predict resistance to androgen deprivation therapy [12]. Because many microbiome effects are mediated through host immunity, immune and inflammatory profiling is essential. Circulating markers such as LPS, IL-6, and TNF-α reflect systemic inflammation, while evaluation of immune cell infiltration and macrophage polarization within the tumor microenvironment provides local context [86]. These assessments must also account for confounding factors, including antibiotic exposure, which is consistently associated with reduced immunotherapy efficacy, and dietary patterns that strongly shape microbial composition and metabolic output. Longitudinal monitoring will enable therapies to be adapted as microbial and metabolic states evolve over time.

Interventions guided by these insights are advancing across multiple domains. Fecal microbiota transplantation has shown promise in restoring immunotherapy sensitivity, while probiotics, synbiotics, and dietary modulation can selectively enrich beneficial taxa. Supplementation with *A. muciniphila*, *Bifidobacterium*, or *C. butyricum* enhances checkpoint inhibitor activity in preclinical models, and dietary strategies such as omega-3 supplementation or reduced choline intake represent accessible personalized interventions. Advances in synthetic biology allow engineered microbes to deliver therapeutic molecules directly within tumors, while selective antimicrobials and metabolic pathway inhibitors can suppress harmful microbial activities without disrupting the broader ecosystem. Integration of host and microbial genetics offers another layer of personalization, as germline polymorphisms influence microbial composition and microbial gene repertoires determine functional capacity, including equol production from soy isoflavones.

These strategies can be particularly impactful in prostate cancer, an immunologically “cold” tumor. Clinical trials are now evaluating fecal microbiota transplantation combined with pembrolizumab, building on encouraging results from melanoma [80]. Probiotics such as *A. muciniphila*, *Bifidobacterium*, and *C. butyricum* enhance anti-tumor immunity in preclinical prostate cancer models, and metabolites including inosine and SCFAs are being explored as adjuvants to improve therapeutic response. Beyond immunotherapy, microbiome modulation may enhance the effectiveness of androgen deprivation and chemotherapy by reducing androgen-producing bacteria, restoring microbial balance, and suppressing inflammatory pathways that promote resistance.

Insights from other malignancies reinforce the broader clinical relevance of microbiome-based interventions. The microbiome also acts as a direct driver of carcinogenesis: Fusobacterium nucleatum and colibactin-producing *E. coli* promote colorectal cancer through genotoxicity, Helicobacter pylori remains central to gastric cancer development, and intratumoral bacteria in pancreatic cancer directly inactivate chemotherapy [85,87]. Microbial metabolites add further complexity, with butyrate protective in colorectal cancer yet immunosuppressive in other contexts, TMAO oncogenic in colorectal and liver cancers yet supportive of immunotherapy in breast and pancreatic cancers, and inosine consistently enhancing T-cell activation across multiple tumor types [88].

These findings establish the gut microbiome as a central and targetable hub in oncology. The key challenges ahead include standardizing protocols for microbiome-based interventions, optimizing donor and strain selection, defining therapeutic dosing, and ensuring reproducibility across trials. Future research must integrate multi-omics approaches to move from correlation to mechanism and to enable precise patient stratification. By embedding microbial, metabolic, and genetic insights into clinical decision-making, microbiome-targeted strategies are positioned to become a cornerstone of precision oncology, offering new opportunities to enhance efficacy, overcome resistance, and improve outcomes across cancer types.

## 5. Conclusions

The gut microbiome is emerging as an important and modifiable contributor to prostate cancer biology. Although mechanistic and preclinical studies provide compelling support for a functional gut–prostate axis, translation to clinical practice remains limited by heterogeneity in study design, confounding environmental factors, and incomplete causal understanding. Ongoing clinical trials evaluating dietary interventions, probiotics, antibiotics, and fecal microbiota transplantation will be critical in defining the therapeutic relevance of microbiome modulation. Future progress will depend on standardized microbiome profiling, integration of multi-omics approaches, and longitudinal studies capable of distinguishing causal microbial drivers from disease-associated signatures. As understanding of the gut–prostate axis deepens, microbiome-centered strategies have the potential to enhance the efficacy of existing therapies, overcome resistance, and contribute to precision oncology approaches in prostate cancer. These strategies may ultimately expand the therapeutic landscape and improve outcomes for patients across the disease spectrum.

## Figures and Tables

**Table 1:** Clinical trials of gut microbiome–based interventions in prostate cancer.

Study ID	Intervention	Population/Disease Setting	Primary Endpoint	Key Secondary Endpoints	Phase/Design
NCT04116775	Fecal microbiota transplantation (FMT) + pembrolizumab + enzalutamide	mCRPC resistant to enzalutamide + pembrolizumab	PSA50 response after FMT and pembrolizumab retreatment	PSA progression, radiographic response, overall survival	Phase 2
NCT03934827	MRx0518 (live biotherapeutic)	Solid tumors awaiting surgical resection, including PCa	Safety and tolerability of MRx0518	Changes in tumor immune microenvironment	Interventional (early phase)
NCT06816597	Abiraterone acetate + dexamethasone + metronidazole	Metastatic prostate adenocarcinoma	PSA30 response over 24 weeks	PSA50 response, progression-free survival, toxicity	Phase 2
NCT06536374	Trimethoprim 150 mg daily for 3 months	Advanced PCa receiving ADT	Safety (≥Grade 3 adverse events)	PSA levels, folate levels, microbiome and metabolomic changes	Phase 2
NCT03987903	Grape seed extract (300 mg/day for 1 year)	Asymptomatic, non-metastatic PCa with rising PSA	Increase in PSA doubling time ≥ 30%	PSA velocity, gut microbiome changes, metabolomics	Phase 2
NCT05850182	Personalized diet and physical activity intervention	Metastatic PCa receiving ADT	Feasibility (initiation and retention rates)	PSA progression, quality of life, fatigue, microbiome composition, lipid profile, body composition	Pilot study
NCT05590624	Mediterranean diet (low carbohydrate, low fat)	High suspicion or confirmed localized PCa	Changes in non-malignant prostate tissue metabolism	Systemic biomarkers, gut microbiome diversity, dietary compliance	Crossover study
NCT06126731	Antibiotics (amoxicillin, metronidazole, ciprofloxacin, vancomycin) + enzalutamide	mCRPC	Safety and tolerability of antibiotic combinations	PSA response, progression-free survival, tumor genomic evolution, immune response	Phase 1/2
NCT04985655	Mediterranean diet (6 days/week for 4 weeks) prior to prostatectomy	Intermediate risk localized prostate adenocarcinoma	Feasibility of dietary intervention	Diet compliance, metabolic parameters, fecal microbiome changes, Cav-1–sphingolipid signature	Single-group assignment
NCT06242509	Plasma and stool microbiome profiling (*A. muciniphila*)	mCRPC receiving next-generation hormonal therapy	Abundance of fecal *A. muciniphila* after 1 month	PSA progression-free survival, microbiome diversity, anti-Akkermansia IgG/IgA levels	Observational cohort
NCT05802121	Apple cider vinegar (acetate supplementation) vs. placebo	Metastatic castration-sensitive PCa initiating ADT	Change in fecal *A. muciniphila* abundance	Metabolic and bone health parameters, PSA levels, insulin resistance (HOMA-IR)	Early Phase 1

## Data Availability

No new data were created or analyzed in this study. Data sharing is not applicable to this article.

## Abbreviations

The following abbreviations are used in this manuscript:

ADT	androgen deprivation therapy
AKT	protein kinase B
AR	androgen receptor
ARKO	androgen receptor knockout
ATBC	alpha-tocopherol, beta-carotene cancer prevention study
BPH	benign prostatic hyperplasia
CCL20	C–C motif chemokine ligand 20
CCR6	C–C chemokine receptor 6
CRPC	castration-resistant prostate cancer
CTLA-4	cytotoxic T-lymphocyte–associated protein 4
DHEA	dehydroepiandrosterone
DHT	dihydrotestosterone
EPA	eicosapentaenoic acid
FMT	fecal microbiota transplantation
HDAC	histone deacetylase
HFD	high-fat diet
HMOX1	heme oxygenase 1
HOMA-IR	homeostatic model assessment of insulin resistance
HSPC	hormone-sensitive prostate cancer
ICIs	immune checkpoint inhibitors
IGF-1	insulin-like growth factor 1
IGF-1R	insulin-like growth factor 1 receptor
IgA	immunoglobulin A
IgG	immunoglobulin G
IL-6	interleukin 6
LPS	lipopolysaccharide
MAG-EPA	monoacylglyceride eicosapentaenoic acid
MAPK	mitogen-activated protein kinase
MDA	malondialdehyde
mCRPC	metastatic castration-resistant prostate cancer
mCSPC	metastatic castration-sensitive prostate cancer
NF-κB	nuclear factor kappa B
ORX	orchidectomy
PB	prebiotic/probiotic blend
PCa	prostate cancer
PD-1	programmed cell death protein 1
PD-L1	programmed death-ligand 1
PI3K	phosphoinositide 3-kinase
PLCO	prostate, lung, colorectal, and ovarian cancer screening trial
PRS	phytochemical-rich supplement
PSA	prostate-specific antigen
RM1	RM-1 prostate cancer model
ROS	reactive oxygen species
SCFA	short-chain fatty acid
STAT3	signal transducer and activator of transcription 3
Th1	T helper 1
Th17	T helper 17
TLR3	toll-like receptor 3
TLR4	toll-like receptor 4
TMA	trimethylamine
TMAO	trimethylamine N-oxide
TME	tumor microenvironment
TNF-α	tumor necrosis factor alpha
TRAMP	transgenic adenocarcinoma of the mouse prostate
Treg	regulatory T cell
